# Supplementary material for: Rare variant analyses validate known ALS genes in a multi-ethnic population and identifies ANTXR2 as a candidate in PLS
Source: BMC Genomics. 2024 Jun 29;25:651. doi: 10.1186/s12864-024-10538-1 (PMC11218304; doi:10.1186/s12864-024-10538-1)
Supplement: Supplementary file 1 — Supplementary Material 1. [file 12864_2024_10538_MOESM1_ESM.pdf]

Supplemental Figures and Tables

Supplemental Figures

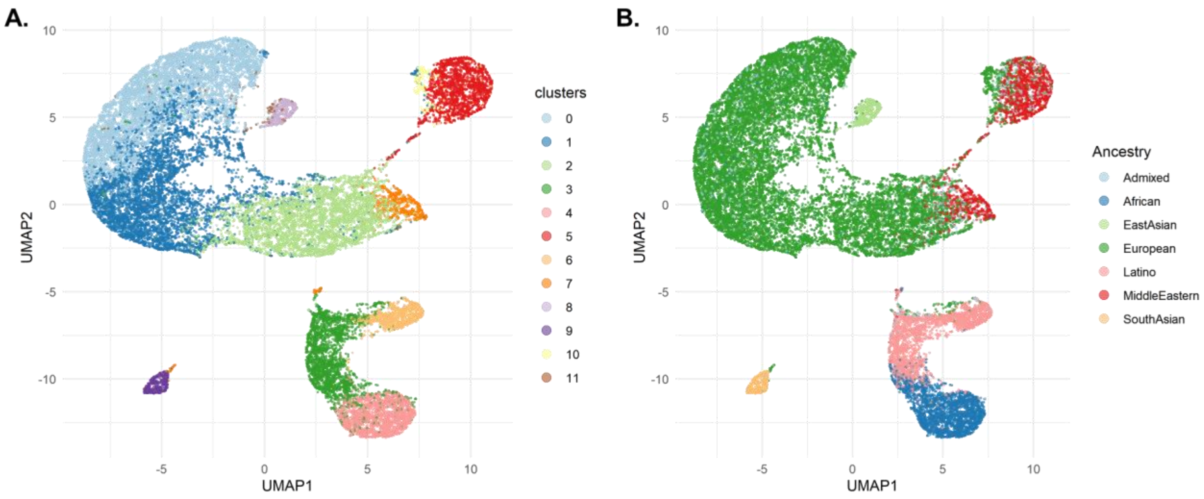

Supplemental Figure 1. **UMAP plot of ALS participants** (A) Cluster assignment (B) Predicted ancestry

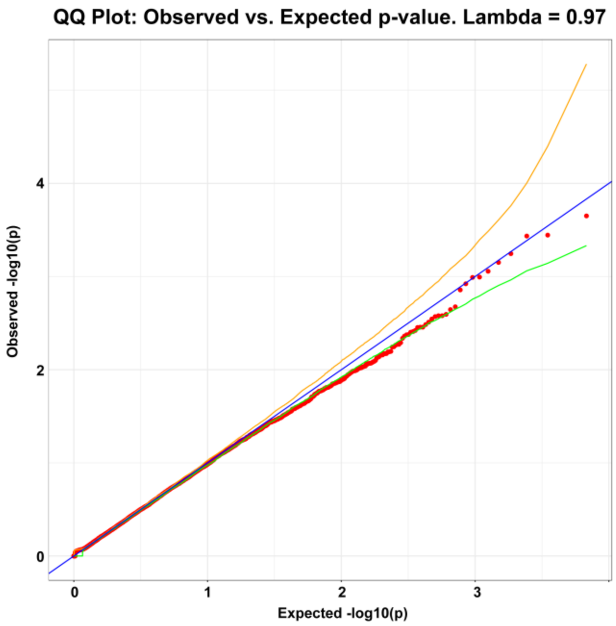

Supplemental Figure 2. Q-Q plots of gene level collapsing in a Synonymous model. Yellow and green lines indicate the 2.5th and 97.5th percentile of expected p-values, respectively. The genomic inflation factor, lambda (λ), is 0.97 indicating no inflation. We generated p values from the exact two-sided Cochran-Mantel-Haenszel (CMH) test by gene by cluster to indicate a different carrier status of affected individuals in comparison to control individuals.

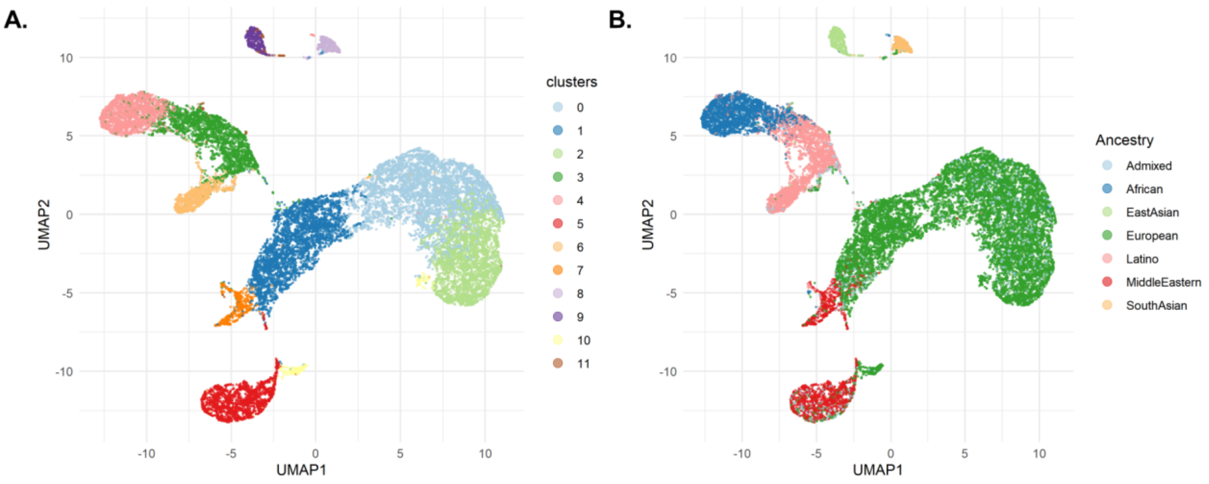

Supplemental Figure 3. **UMAP plot of PLS participants** (A) Cluster assignment (B) Predicted ancestry

Supplemental Tables

Supplemental Table 1. Sequencing kits used for cohort

Sample Type	Capture Kit	Cases	Controls
Exome	Agilent All Exon kits	0	1,272
	Nimblegen SeqCap EZ Exome Enrichment kits	2,185	11,201
	IDT Exome Enrichment panel	51	8,498
Genome		4,734	1,553
Total		6,970	22,524

Supplemental Table 2. Description of collapsing models

	Synonymous	PTV	Missense&PTV	Missense&PTV subRVIS
Inheritance	Dominant	Dominant	Dominant	Dominant
Functions	Synonymous	PTV	Missense + PTV	Missense + PTV
LOO MAF	0.001	0.001	0.001	0.001
External MAF	0.001	0.001	0.001	0.001
Additional Filters				subRVIS Domain Percentage <25

Supplemental Table 3. Cluster sizes for ALS cohort

Cluster	Ancestry	Cases	Controls	Ratio
0	European1	2,620	4,792	0.55
1	European2	2,502	3,989	0.63
2	European3	922	3,868	0.24
3	Latino1	104	2,788	0.037
4	African1	204	2,102	0.097
5	MiddleEastern1	294	1,924	0.15
6	Latino2	110	1,068	0.1
7	MiddleEastern2	86	669	0.13
8	EastAsian1	35	496	0.071
9	SouthAsian1	40	457	0.088
10	European4	36	265	0.14
11	Admixed1	17	106	0.15
Total		6,970	22,524	0.31

Supplemental Table 4. Phenotypes of control participants

Control Phenotype	Number of Participants
Kidney and urological disease	8,416 (37.36%)
Healthy family member of a different study	6,297 (27.96%)
Control of different study	3,156 (14.01%)
Epilepsy	2,892 (12.84%)
Obsessive compulsive disorder	721 (3.2%)
Pulmonary disease	403 (1.79%)
Hematological disease	256 (1.13%)
Schizophrenia	161 (0.71%)
Infectious disease	107 (0.48%)
Liver disease	82 (0.36%)
Primary immune deficiency	33 (0.15%)
Total	22,524

Supplemental Table 5. Cluster sizes for PLS cohort

Cluster	Ancestry	Cases	Controls	Ratio
0	European1	90	4,792	0.017
1	European2	19	3,989	0.0047
2	European3	41	3,868	0.012
3	Latino1	1	2,788	0.00039
4	African1	3	2,102	0.0013
5	MiddleEastern1	10	1,924	0.0052
6	Latino2	3	1,068	0.0027
7	MiddleEastern2	1	669	0.0016
8	SouthAsian1	2	496	0.004
9	EastAsian1	1	457	0.002
10	European4	1	265	0.0028
11	Admixed1	0	106	0
Total		172	22,530	0.0076

Supplemental Table 6. Description of PLS participants with *ANTXR2* PTV Qualifying Variants

ID	Sex	Age Range at Onset	Phenotype	Location Hg19	Effect	Depth	MAF	Alleles in Gnomad 2.1
**PLS1**	M	66-70	Leg onset	4-80990653-A-G	p.Met1?	39x	0.44	0
**PLS2**	F	41-45	Leg onset	4-80905972-C-T	c.1086+1G>A	29x	0.48	0
**PLS3**	M	61-65	Leg onset	4-80905984-CA-C	p.Ala359fs	20x	0.35	3
**Ctrl1**	F	Unk	OCD	4-80957125-C-T	c.697+1G>A	18x	0.67	1
**Ctrl2**	M	Unk	Kidney disease	4-80977127-TA-T	p.Ser113fs	20x	0.6	0
