# Supplementary material for: Rare variant analyses validate known ALS genes in a multi-ethnic population and identifies ANTXR2 as a candidate in PLS
Source: BMC Genomics. 2024 Jun 29;25:651. doi: 10.1186/s12864-024-10538-1 (PMC11218304; doi:10.1186/s12864-024-10538-1)
Supplement: Supplementary file 3 — Supplementary Material 3. [file 12864_2024_10538_MOESM3_ESM.docx]

**Supplementary Information**

**Study population**

All samples and data came from participants that provided written, informed consent for genetic studies that had been IRB-approved at each contributing center. The study cohort includes participants from the Genomic Translation for ALS Care (GTAC study), the Columbia University Precision Medicine Initiative for ALS, the ALS COSMOS Study Group, the PLS COSMOS Study Group, the New York Genome Consortium, and the ALS Sequencing Consortium (IRB-approved genetic studies from Columbia University Medical Center, including the Coriell NINDS repository), University of Massachusetts at Worchester, Stanford University (including samples from Emory University School of Medicine, the Johns Hopkins University School of Medicine, and the University of California, San Diego), Massachusetts General Hospital Neurogenetics DNA Diagnostic Lab Repository, Duke University, McGill University (including contributions from Saint-Luc and Notre-Dame Hospital of the Centre Hospitalier de l’Université de Montréal [CHUM], [University of Montreal]), Gui de Chauliac Hospital of the CHU de Montpellier (Montpellier University), Pitié Salpêtrière Hospital, Fleurimont Hospital of the Centre Hospitalier Universitaire de Sherbrooke (CHUS) (University of Sherbrooke), Enfant Jésus Hospital of the Centre hospitalier affilié universitaire de Québec (CHA) (Laval University), Montreal General Hospital, Montreal Neurological Institute and Hospital of the McGill University Health Centre, the University of Edinburgh Scotland, and Washington University in St. Louis (including contributions from Houston Methodist Hospital, Virginia Mason Medical Center, University of Utah, and Cedars Sinai Medical Center). Participants were determined to have ALS or PLS by neuromuscular specialists at tertiary motor neuron disease care centers with expertise in distinguishing between the two. ALS diagnoses were based on the El Escorial Criteria in all cases. For PLS, explicit criteria requiring >3 years of symptoms without conversion to ALS were used for 79 of the 172 PLS participants (1). The criteria used for the remaining PLS diagnoses were not available.

Controls were selected from >100,000 whole-exome or -genome sequenced individuals housed in the IGM Data Repository. Individuals with known neurodegenerative disease were excluded. However, none of the controls were screened for neurodegenerative disease. All participants consented to the use of DNA in genetic research.

**References**

1. Mitsumoto H, Factor-Litvak P, Andrews H, Goetz RR, Andrews L, Rabkin JG, et al. ALS Multicenter Cohort Study of Oxidative Stress (ALS COSMOS): study methodology, recruitment, and baseline demographic and disease characteristics. Amyotroph Lateral Scler Frontotemporal Degener. 2014;15(3-4):192-203.
